# Optimizing Intradermal Administration of Cryopreserved *Plasmodium falciparum* Sporozoites in Controlled Human Malaria Infection

**DOI:** 10.4269/ajtmh.15-0341

**Published:** 2015-12-09

**Authors:** Kirsten E. Lyke, Matthew B. Laurens, Kathy Strauss, Matthew Adams, Peter F. Billingsley, Eric James, Anita Manoj, Sumana Chakravarty, Christopher V. Plowe, Ming Lin Li, Adam Ruben, Robert Edelman, Michael Green, Tina J. Dube, B. Kim Lee Sim, Stephen L. Hoffman

**Affiliations:** Center for Vaccine Development, University of Maryland School of Medicine, Baltimore, Maryland; Howard Hughes Medical Institute, University of Maryland School of Medicine, Baltimore, Maryland; Sanaria Inc., Rockville, Maryland; Protein Potential LLC, Rockville, Maryland; Centers for Disease Control and Prevention, Atlanta, Georgia; The EMMES Corporation, Rockville, Maryland

## Abstract

Controlled human malaria infection (CHMI) is a powerful tool to evaluate malaria vaccine and prophylactic drug efficacy. Until recently CHMI was only carried out by the bite of infected mosquitoes. A parenteral method of CHMI would standardize *Plasmodium falciparum* sporozoite (PfSPZ) administration, eliminate the need for expensive challenge facility infrastructure, and allow for use of many *P. falciparum* strains. Recently, intradermal (ID) injection of aseptic, purified, cryopreserved PfSPZ was shown to induce *P. falciparum* malaria; however, 100% infection rates were not achieved by ID injection. To optimize ID PfSPZ dosing so as to achieve 100% infection, 30 adults aged 18–45 years were randomized to one of six groups composed of five volunteers each. The parameters of dose (1 × 10^4^ versus 5 × 10^4^ PfSPZ total dose per volunteer), number of injections (two versus eight), and aliquot volume per ID injection (10 μL versus 50 μL) were studied. Three groups attained 100% infection: 1 × 10^4^ PfSPZ in 50 μL/2 doses, 1 × 10^4^ PfSPZ in 10 μL/2 doses, and 5 × 10^4^ PfSPZ in 10 μL/8 doses. The group that received 5 × 10^4^ PfSPZ total dose in eight 10 μL injections had a 100% infection rate and the shortest prepatent period (mean of 12.7 days), approaching the prepatent period for the current CHMI standard of five infected mosquitoes.

## Introduction

Controlled human malaria infection (CHMI) studies have been used as a powerful tool to evaluate malaria vaccine and prophylactic drug efficacy since the mid-1980s. CHMI data inform the decision for or against further clinical development of candidate vaccines, including whether to test in a pediatric population. Although experience is limited, both negative and positive results of CHMI trials conducted in malaria-naïve individuals have been predictive of field efficacy results to date.^1^,^2^ No vaccine has demonstrated reproducible protective efficacy in malaria-endemic settings without first showing efficacy against CHMI.^3^ Prevention of infection in a proportion of malaria-naïve persons (as with the RTS,S/AS01 vaccine)^4^ is considered evidence of partial efficacy, and reason for advancement of vaccine candidates along clinical development pathways. Because of the critical importance of CHMI in assessing candidate malaria vaccines, efforts have aimed to further standardize the model across institutions.

Obstacles to a fully standardized CHMI for assessing new malaria vaccines and drugs include variability in infection rates between batches of mosquitoes and varying infectivity during the 7-day window when the mosquitoes are most suitable for use in CHMI. These differences can lead to inconsistent prepatent periods (time from CHMI to patent parasitemia) and occasional failures of volunteers to become infected. Many factors restrict access to CHMI to only a few institutions, including the expertise and cost required to maintain mosquito colonies, and the need for secure facilities for CHMI studies, although these limitations can be partly overcome by shipping infected mosquitoes to distant sites. Additional issues include the reactogenicity of mosquito bites due to injection of mosquito saliva and the potential safety concerns associated with using non-sterile mosquitoes. These various obstacles provided a rationale for the development of PfSPZ Challenge^®^ (Sanaria, Inc., Rockville, MD), a good manufacturing practice (GMP) product manufactured by Sanaria Inc. that consists of aseptic, cryopreserved, infectious *Plasmodium falciparum* sporozoite (PfSPZ) that meet all regulatory requirements for purity, potency, and consistency, and that are suitable for parenteral injection as a potential replacement for mosquito bites for performing CHMI.

The first trial of PfSPZ Challenge was conducted at the Radboud University Nijmegen Medical Center (RUNMC) in the Netherlands in 2010–2011. Eighteen participants were randomized to intradermal (ID) administration of 2.5 × 10^3^, 1 × 10^4^, and 2.5 × 10^4^ aseptic, purified, cryopreserved PfSPZ, in two doses of 50 μL each, and five of six subjects became infected with *P. falciparum* malaria at all three dose levels.^5^ These results were replicated in malaria-experienced volunteers in Tanzania where 11 of 12 volunteers receiving 1 × 10^4^ PfSPZ divided into two doses of 50 μL and 10 of 11 evaluable volunteers receiving 2.5 × 10^4^ PfSPZ divided into four doses of 10 μL developed *P. falciparum* parasitemia after ID injection of PfSPZ Challenge.^6^ The prepatent periods of the three groups in Nijmegen were 13.0, 12.7, and 13.0 days, respectively, and 15.4 and 13.5 for the two groups in Tanzania. Thus in none of the five groups in these two studies did all research subjects become infected, and in none was the prepatent period as determined by microscopy equivalent to the 10.5–12 days typical of CHMI by five mosquito bites.

The results of these two ID CHMI studies posed several questions regarding the administration of PfSPZ Challenge. Were too few PfSPZ administered? Was aliquot volume too great? Would an increase in number of injections improve efforts to mimic the mechanics of mosquito biting, which involve repetitive probing and injection of microaliquots of PfSPZ while biting? The anatomic location of PfSPZ injection or the modalities of delivery, such as intramuscular (IM), intravenous (IV), or a multi-needle microdose injectable device may also be important variables. Based on a design that manipulated three parameters (dose, injection number, and volume), coupled with an assessment of the kinetics and quantification of parasitemia by polymerase chain reaction (PCR), the result of ID administration of PfSPZ was further evaluated.

## Methods

### Objectives.

This study was designed as a dose-ranging, randomized, prospective Phase I cohort study, assessing whether ID administration of cryopreserved PfSPZ of the NF54 isolate of *P. falciparum* (PfSPZ Challenge) results in prepatent periods and infection rates similar to those obtained via the bites of mosquitoes. The main objectives were to determine safety and tolerability of parenterally administered CHMI and to discern the optimal dose required to achieve 100% infection in adult subjects. The parameters of 1) dose, 2) number of injections, and 3) aliquot volume were studied.

### Study population and design.

The clinical study was conducted at the Center for Vaccine Development, at the University of Maryland School of Medicine (UMB) in Baltimore, MD. Thirty malaria-naive adults aged 18–45 years were randomized into one of six groups to receive a total dose of 1 × 10^4^ (four groups) or 5 × 10^4^ PfSPZ (two groups) of PfSPZ Challenge. The total dose was split into either two or eight PfSPZ injections administered parenterally via ID injection in either 10 μL (two groups of 1 × 10^4^ and two groups of 5 × 10^4^ PfSPZ) or 50 μL aliquots (two groups of 1 × 10^4^ PfSPZ) during a single administration session with a total of five subjects per group (Table 1). The lower limit of injectate by needle and syringe is currently ∼5–10 μL. As 50-μL injections in two divided doses did not achieve 100% infection,^5^ this study was weighted toward an injectate volume of 10 μL. Although increasing the volume of injectate above 50 μL would likely contribute to tissue destruction, it might also lead to sporozoites being trapped within the injectate and unable to be distributed systemically. We postulated the combination of multiple injections and microaliquots of sporozoites might simultaneously enhance tissue destruction while enabling sporozoite penetration into the capillary system allowing for hepatic delivery. An upper limit of eight injections balanced the need to increase injection number while preserving practicality in a clinical trial setting. Participants were randomized by an online allocation sequence generated by the EMMES Corporation (Rockville, MD). Study personnel were blinded to group assignment. Participants were previously screened for good health, including laboratory testing for hepatitis B and C, human immunodeficiency virus, and for pregnancy testing in females of childbearing potential. Baseline complete blood counts, creatinine, glucose, aspartate aminotransferase (AST), and alanine aminotransferase (ALT) were screened and only volunteers with normal values were enrolled. In addition, volunteers with significant cardiovascular risk were excluded (i.e., > 10%, 5-year risk),^7^ including evaluation by gender and age for systolic blood pressure (mmHg), smoking status (current versus past or never), body mass index (kg/mm^2^), reported diabetes status, and current treatment of raised blood pressure. A 12-lead electrocardiogram (ECG) was performed and read by a staff cardiologist. Exclusion criteria included known history of malaria infection, long-term residence (> 5 years) in a malaria-endemic area, travel to a malaria-endemic area within the previous 6 months, the presence of hemoglobin S by electrophoresis, and splenectomy.

### Dosage, preparation, and administration of study product.

The investigational PfSPZ Challenge product was cryopreserved, in liquid nitrogen vapor phase at −150 to −196°C, as a suspension of aseptic, purified, replication-intact PfSPZ formulated in cryoprotectant. The challenge product was administered ID to the deltoid area of the upper arm within 30 minutes of reconstitution by a study nurse unblinded to the number and volume of assigned injections. After randomization to two or eight injections, half were administered in the right arm and half to the left arm, separated by a width of approximately 1 cm when eight injections were given.

### Post-administration assessment.

Participants were monitored for 30 minutes after ID CHMI and asked to maintain symptom diaries until Day 7. They were evaluated in clinic on Days 5–7 and admitted to an in-patient ward on Day 8 before the expected time of blood stage parasitemia. Daily histories, vital signs, targeted physical examinations, blood smears, and real-time quantitative PCR (qPCR) assays were performed. Asymptomatic individuals were provided with cell phones, allowed to leave the ward during the day, and return in the evenings for evaluation. Symptomatic or parasitemic individuals remained on the ward under clinical supervision. Inpatient analysis occurred from Days 8 to 18 or until documentation of parasitemia and receipt of 1,500 mg chloroquine (CQ) base as standard first-line therapy over 48 hours (600 mg base/1,000 mg salt at time 0 followed by 300 mg oral base/500 mg salt at hours 6, 24, and 48) and two negative blood smears ≥ 12 hours apart. A third negative smear, ≥ 12 hours after the previous two negative smears, was documented as an outpatient to affirm malaria cure. As part of a sub-study to evaluate the kinetics of CQ single-dose therapy, CQ and its highly active metabolite, desethylchloroquine (DCQ), levels in blood and urine were measured in a subset of parasitemic volunteers on Days 0, 2, 7, 14, 21, and 28 post-malaria diagnosis. The technique for detecting CQ and DCQ has been validated in blood^8^ and urine^9^ and was performed at the Centers for Disease Control (CDC). Volunteers who did not acquire malaria were terminally treated with atovaquone-proguanil (Malarone^®^; GlaxoSmithKline, Research Triangle Park, NC) starting on Day 28 post-CHMI. Unsolicited adverse events were recorded for 56 days after the CHMI event.

### Assessment of safety and tolerability.

All adverse events were graded for 1) severity (mild, moderate, severe, or life threatening) and 2) relatedness (associated or not associated to the study product). Specific severity scales were provided for fever (mild, > 99.5–100.4°F; moderate, > 100.4–102.2°F; and severe, > 102.2°F), and erythema (mild, 2.5–5 cm; moderate, 5.1–10 cm; and severe, > 10 cm). The local injection site was assessed and general solicited symptoms or signs evaluated until Day 14 post-CHMI. Local reactions that persisted beyond Day 2 were recorded as adverse events based on previous site experience that erythema, pruritus, and induration are normal responses to mosquito bites within two days of exposure. Solicited systemic symptoms related to the malaria challenge and before malaria parasitemia were recorded until Day 7 post-CHMI (Table 2) and those related to blood stage malaria began on Day 8 post-CHMI (Table 3) and continued for the duration of the volunteer follow-up or until malaria diagnosis. A comparison was made assessing historical results from our site with a traditional CHMI using aseptically reared *Anopheles stephensi* mosquitoes infected with the same strain of *P. falciparum* (NF54) and cultured in the same laboratory facility to eliminate confounding variables (Table 3). Any other signs or symptoms were considered unsolicited until Day 28. Serious adverse events were monitored until Day 56 after CHMI.

### Malaria diagnostics.

#### Blood smears.

Beginning on Day 6 post-CHMI, daily blood smears were performed to monitor for the presence of blood stage parasites using techniques previously described.^10^,^11^ If participants developed signs or symptoms of malaria, bloods smear interval preparation decreased to every 8–12 hours until a diagnosis was established. Two trained investigators, blinded to randomization results, examined approximately 0.5 μL of blood using the 100× oil immersion lens of calibrated microscopes. This was doubled to approximately 1.0 μL for symptomatic individuals. Parasites were quantified per microliters. For positive smears, or if questions or discrepancies arose, an expert reader verified positivity and quantified parasite burden. The minimum criterion for acceptance of a positive smear was identification of two unquestionable *P. falciparum* parasites. All therapeutic decisions were based on positive blood smear results.

#### Real-time quantitative DNA polymerase chain reaction.

qPCR was performed on 0.5 mL of venous blood collected contemporaneously with blood smears using published methods with minor modifications.^12^ Samples were blinded and assays were run daily. Deoxyribonucleic acid (DNA) extraction was performed with a QIAamp DNA Mini kit (Qiagen, Hilden, Germany) modified to accommodate 0.5 mL whole blood. Extraction efficiency was verified by the addition and subsequent qPCR analysis of an Extraction Control with a proprietary probe and primers (DNA Extraction Control 560; Bioline, Taunton, MA). PCR primers were based on the published sequence of the highly conserved,^13^ stage-specific^14^ *P. falciparum* 18S ribosomal RNA gene. Primer sequences corresponded to the sequence of the NF54 strain and were as follows: Forward— 5′- GTA ATT GGA ATG ATA GGA ATT TAC AAG GT -3′, Reverse— 5′- TCA ACT ACG AAC GTT TTA ACT GCA AC -3′, and Probe— 5′-FAM GAA CGG GAG GTT AAC AA MGB-3′. Each sample was run in duplicate along with “no template controls” and against standards at the following *P. falciparum* concentrations: 600,000; 200,000; 60,000; 20,000; 6,000; 2,000; 600; 200; 60; and 20 parasites/mL diluted in human whole blood. DNA amplification was done as previously described,^10^,^11^ but with the preincubation step extended to 15 minutes, on an Applied Biosystems 7300 Real Time PCR System (Foster City, CA). Data were analyzed using the Applied Biosystems 7300 Absolute Quantification Software and were compared with blood smear results.

#### Antibody assays.

*Plasmodium falciparum* antibodies to circumsporozoite protein (CSP), merozoite surface protein 1 (MSP1), and erythrocyte-binding antigen-175 (EBA-175) by enzyme-linked immunosorbent assay (ELISA), and to sporozoites by the immunofluorescence assay (IFA) were assessed in sera as previously described^15^ on the day of injection of PfSPZ Challenge and 28 days later. Antibodies against *P. falciparum* Exported Protein 1 (PfEXP1) were assessed in ELISAs against recombinant PfEXP1 (rPfEXP1) expressed in *Pichia pastoris.* The gene encoding rPfEXP1 expressed did not contain the signal sequence (residues 1–19) and had site-specific mutagenesis of amino acids V87 to N and P160 to T. The 96-well ELISA plates were coated with rPfEXP-1 at a concentration of 0.5 ng/μL in a volume of 50 μL/well (25 ng/well). Plates were incubated overnight at 4°C. Plates were washed thrice with 1X Imidazole-based wash solution containing 2 mM imidazole, 160 mM NaCl, 0.02% Tween 20, 0.5 mM EDTA and blocked with 1% bovine serum albumin (BSA) blocking buffer (KPL) containing 1% non-fat dry milk for 1 hour at 37°C. Further processing of plates and addition of sera samples for assessments were identical to that described for PfEBA-175.^15^ The plates were read with a SPECTRAmax^®^ *PLUS*^384^ microplate spectrophotometer (Molecular Device, Sunnyvale, CA) at 405 nm. The data collected using Softmax 5.0 were fit to a 4-parameter logistic curve, and the serum dilution at which the optical density was 1.0 (OD 1.0) was calculated. A positive control (sera from malaria-endemic area, Kenyan Blood Bank) and a negative control (sera from naïve individuals, Lifeblood, Memphis, TN) were always included. ELISA responses were arbitrarily considered positive if the difference between the serum dilution at which the optical density was 1.0 (OD 1.0) on Day 28 and preinjection of PfSPZ Challenge was ≥ 50, and the ratio of postinjection to preinjection OD 1.0 was ≥ 2.5.

To assess antibodies to *P. falciparum* asexual erythrocytic stage parasites by IFA, wells of Cel-Line^®^ slides (Thermo Scientific, Waltham, MA) were spotted with 20 μL of a > 0.5% asexual stage *P. falciparum* culture in 2% BSA (Sigma Aldrich), and air dried for 18 hours before use. Sera samples for assessment were diluted in 10% Normal Mouse Serum (KPL Cat no. 71-18-01). Preimmune sera samples were added at a single dilution of 1:50 and the postinjection samples were added at 2-fold dilutions starting at 1:50. Further processing and assessment steps were as previously described.^15^ The positive control sera samples were a pool of sera from human volunteers immunized with recombinant PfMSP1 and with recombinant PfEBA-175 separately. Human negative control sera sample was pooled sera from malaria naïve humans (Key Biologics LLC, Memphis, TN). The end point titer was defined as the last serum dilution at which fluorescence intensity was higher than the fluorescence intensity of the preimmune sera. A postimmunization serum sample was considered positive if it had fluorescence at a dilution of 1:50 or higher, and the preimmunization serum from that volunteer was negative at 1:50.

#### CQ and DCQ kinetic analysis.

The CQ and DCQ blood and urine kinetic sub-study was designed to assess the drug kinetics of CQ over the course of 4 weeks following standard CQ dose administration. The goal was to use measured levels to model whether directly observed CQ dosing at longer treatment intervals is a feasible option compared with standard weekly prophylactic doses in preventing parasitemia following parenterally administered PfSPZ products. CQ and DCQ levels in blood/urine were measured in a subset of 18 subjects who acquired malaria and had successfully completed CQ administrations as well as follow-up visits that aligned most closely with days 0, 2, 7, 14, 21, and 28 post-malaria episode. Participation was limited to 18 volunteers at six time points by contractual agreement and supply of available reagents. The technique for detecting CQ and DCQ was validated in blood^8^ and urine^9^ and was performed by collaborators at the CDC. Subject participation began after the diagnosis of peripheral parasitemia by microscopy. Noncompartmental CQ kinetics analysis was used to estimate the slope of the elimination phase, elimination half-life, maximum concentration, time of maximum concentration, and area under the curve. All pharmacokinetic (PK) parameters, with the exception of area under the concentration–time curve from time zero to final sample (AUC_last_), were estimated with respect to the fourth dose of CQ. Samples with concentrations below the lower limit of quantification (LLQ) were not included in the PK parameter estimation. Subjects with fewer than three sample concentrations above the LLQ, or with an *R*^2^ (square of the correlation coefficient) value for elimination phase samples below 0.8, were not included in the analysis. In CQ and DCQ concentration summaries by subject and time point, concentrations below the LLQ were imputed as 0 ng/mL. Elimination half-life was estimated from the slope of the elimination phase, λ_z_. The AUC_last_ was calculated from the first dose to the last sample above the LLQ. The analysis variables consisted of baseline variables, safety variables, efficacy data, and laboratory data. Hours of collected samples and administered doses are calculated from the time of the first dose. PK parameter estimation and statistical analysis were performed in WinNonlin version 6.3 (Certara, Princeton, NJ).

#### Statistics.

The study was designed as a proof-of-concept and was not powered for statistically significant comparisons between groups. Efficacy analysis in this study refers to the efficiency at which 100% infectivity is conferred by each dosing regimen. Each subject's exposure to the randomized quantity, injection number, and aliquot volume was considered a separate Bernoulli trial with “efficacy success” defined as a positive malaria smear within 28 days of exposure. Continuous variables were summarized with standard descriptive statistics, whereas categorical variables were described using Mantel–Haenszel Chi^2^ or Fisher Exact analyses, as appropriate.

The blood-stage parasite multiplication rate was calculated as described in the literature by Bejon and others^16^ and Roestenberg and others^17^ and represents the fold replication of *P. falciparum* parasites in 48 hours. A representation of the multiplication rate was evaluated graphically for each participant and those with a negative slope as well as those with fewer than four qPCR measurements were excluded from the analysis.

## Results

### Study population and CHMI event.

Thirty adults aged 21–44 years (mean: 33 years, 40% female) underwent CHMI in February 2012 by ID administration of 1 × 10^4^ or 5 × 10^4^ PfSPZ in 10 or 50 μL aliquots divided into two or eight injections on the same day (Table 1). The safety, tolerability, and efficacy of the inoculation regimens were evaluated. One volunteer was withdrawn post-CHMI due to a work schedule conflict and early termination anti-malaria therapy was provided (Group A). All volunteers completed four weekly safety follow-ups after treatment of malaria or, terminal treatment in the case of volunteers without parasitemia by Day 28 post-CHMI.

Of the 29 volunteers who completed follow-up through Day 28 post-CHMI, 23 (79.3%) developed patent malaria (Table 4). Two volunteers missed a follow-up day immediately before their malaria diagnosis and their results are removed from group analysis (Volunteer M11UMD012, Group C; and M11UMD060, Group F). Three of the groups (A, C, and F) demonstrated 100% infectivity, which would be expected of the traditional 5-mosquito CHMI. Notably, Groups B and D, which both had a total of eight injections, did not experience higher infectivity nor did the concentration of PfSPZ/μL appear associated with higher infectivity (Tables 1 and 4). In addition, the number of PfSPZ injected (1 × 10^4^ versus 5 × 10^4^) did not play a role in the resulting quantification of parasitemia at the first positive blood smear (14.8 versus 10.1 parasites/μL [range 2–134], *P* = 0.17). These values were comparable to those from prior CHMI studies conducted at our trial site (geometric mean [GM] of 15.7 [range 4–70 parasites/μL]).^10^

Parasitemia by qPCR was detected in all 23 volunteers who developed smear-positive malaria. Combined data from the 23 participants who developed malaria demonstrated that qPCR detected patency with a GM of 60.5 hours before microscopy (95% confidence interval = 46.4–74.6). However, the kinetics of the malaria infection for ID CHMI appeared different from that observed with CHMI by mosquito bite (traditional CHMI). The prepatent period from ID CHMI to first detectable qPCR was 256.8 hours (10.7 days) as compared with 206.4 hours (8.6 days) by traditional CHMI (*P* < 0.001). This delay in detectable parasites was mirrored in the prepatent period for blood smear detection. A mean prepatent period of 13.5 days (range 12–16) by ID CHMI was detected as compared with a prepatent period of 10.9 days by traditional CHMI (*P* < 0.001).^10^ The first detectable qPCR value, however, was not significantly different between ID CHMI and the traditional CHMI (161 versus 193 GM parasites/mL, *P* = 0.40). Parasitemia detection by qPCR was performed daily for diagnostic evaluation compared with more frequent evaluation for kinetic analysis, which limits our interpretation. However, using the method described by Roestenberg and others^5^ and accounting for time in hours, the GM parasite multiplication rate was 9.0 by ID CHMI compared with 13.3 in infectivity controls in a traditional five-mosquito CHMI (unpublished data derived from previous studies conducted at UMB using mosquitoes infected with the 3D7 clone of NF54 strain *P. falciparum*). These minor differences may be of little clinical relevance. By convention, all participants with fewer than four qPCR positive measurements (*N* = 8), were excluded from multiplication rate analysis for a total of 13 evaluable participants. No participants displayed a negative slope. Two volunteers were excluded due to a missing interval sample. The number of parasites detected in the first qPCR cycle of ID CHMI was not significantly different from that detected in the traditional CHMI (85 versus 109 GM parasites/mL, *P* = 0.53).

### Post-administration safety assessment.

Systemic events attributable to the administration of PfSPZ Challenge were ascertained among all subjects through Day 7 post-CHMI (Table 2). Local reactogenicity events from the time of administration were assessed through Day 14. During Days 8–18 post-CHMI through the inpatient stay, systemic reactogenicity events that were expected due to the malaria event were also assessed (Table 3). Safety laboratories were drawn on each day of treatment and subsequent weekly follow-up visits (Days 22, 28, 35, and 42). Five subjects (one in Group B, three in Group D, and one in Group E), who did not develop malaria, were assessed for systemic reactogenicity after inpatient discharge through Day 28 post-CHMI and developed no symptoms with the exception of an unrelated Grade 1 headache. No severe solicited symptoms or signs were noted in volunteers after ID CHMI. Moderate solicited local or systemic events were reported in eight individuals (26.7%) and included five instances of myalgia and four headaches. Mild to moderate reactogenicities were reported most commonly as erythema and injection site tenderness, occurring in a minority of participants. This compared favorably to tabulated signs and symptoms elicited by PfSPZ, delivered by the bite of three aseptic mosquitoes, produced by Sanaria under current GMPs (cGMPs)^11^ with a total of 26.7% of volunteers manifesting a local sign or symptom as compared with 52% of volunteers undergoing CHMI by mosquito bite (8/30 versus 13/25, *P* = 0.09) (Table 2).

As previously described, the kinetics of the parasitemia differed from results documented during traditional CHMI by mosquito bite. Thus, we examined clinical symptoms to determine if differences existed between groups and between the two techniques, Systemic reactogenicity was documented for the period during which malaria would be expected to manifest (Days 8–18 post-CHMI) (Table 3). During this inpatient period, three subjects (10.3%) experienced a severe reaction and 11 subjects (37.9%) experienced moderate reactions. The most frequently reported moderate severity reactions were oral temperature (nine subjects, 31.0%), malaise and myalgia (seven subjects, 24.1%), and chills (six subjects, 20.7%). Severe oral temperature (ranging from 100.4°F to 102.2°F) was reported for two subjects (both in Group F: 5 × 10^4^ total dose of PfSPZ ID in eight injections aliquot into 10 μL volume [i.e., 5 × 10^4^ PfSPZ ID 8 × 10 μL]). Two subjects had severe malaise (Group C: 1 × 10^4^ PfSPZ ID 2 × 10 μL and one in Group F: 5 × 10^4^ PfSPZ ID 8 × 10 μL), one subject had a severe headache (Group C: 1 × 10^4^ PfSPZ ID 2 × 10 μL), and one had severe chills (Group F: 5 × 10^4^ PfSPZ ID 8 × 10 μL). The numbers of subjects by group experiencing a severe systemic reaction are shown in Table 3.

In general, the ID CHMI volunteers experienced less systemic signs and symptoms related to malaria than traditional CHMI comparators. This difference was statistically significant for temperature, chills, headache, and overall symptoms (Table 3). However, the majority of symptoms in the ID CHMI (50–67%) were noted in volunteers who received the high-dose aliquot of 5 × 10^4^ PfSPZ (Groups E and F) providing further evidence that these groups more closely mimicked the traditional CHMI in terms of infectivity, patency, and clinical symptoms.

Laboratory abnormalities were noted in 23 of 23 (100%) participants who developed malaria. Among these, 64 values were graded 1 or higher (53 Grade 1, 7 Grade 2, 2 Grade 3, and 2 Grade 4) with mild AST (61%), ALT (48%), and creatinine elevations (22%) being the most common. Three subjects experienced a Grade 3 or higher clinical laboratory value for bilirubin (peak 1.9 in Group D [1 × 10^4^ PfSPZ ID 8 × 10 μL], and 2.2 and 4.2 mg/dL in Group C [1 × 10^4^ PfSPZ ID 2 × 10 μL]). Laboratory abnormalities peaked during malaria treatment and self-resolved by weekly follow-ups. Transient low hemoglobin, white blood cell counts, and low platelet counts were documented, however, no volunteers experienced Grade 3 thrombocytopenia as previously documented in traditional CHMI studies.^11^ No hypoglycemic events were recorded. All results of ECG testing and serum troponin levels were normal.

Subject M11UMD012 (Group C: 1 × 10^4^ PfSPZ 2 × 10 μL) was noted to be qPCR positive on Day 28 after therapy and was reported and assessed as a Serious Adverse Event bythe sponsor Medical Monitor. Following ID CHMI, a 44-year-old, male volunteer developed the expected outcome of falciparum malaria on Day 15 post-CHMI with a parasite density of 270 parasites/μL by smear. This volunteer missed one interval appointment on the day before detection. He was treated with a total of 3 g of rapid-acting, directly observed atovaquone-proguanil (Malarone) over 3 days. On Day 22 post-CHMI, qPCR and malaria smears were negative. On Day 28 post-CHMI, a routine study qPCR returned positive (295 parasites/mL); however, the malaria smear was negative and the subject was asymptomatic. A repeat qPCR on Day 33 (the day after initial qPCR positive results were forwarded to investigators) post-CHMI was borderline positive (31 parasites/mL, lower limit of detection 20–40), and the subject remained asymptomatic. On Day 35 post-CHMI, the qPCR and malaria smear results were negative. Subsequent malaria smears and qPCR remained negative and no evidence of asexual or replicating stages were found. Terminal CQ treatment was administered per study sponsor request. A *P. falciparum* culture (considered the gold standard for detection of parasites) was performed from blood obtained on Day 28 and remained negative for 4 weeks after incubation.

### Antibody results.

**Figure 1.**
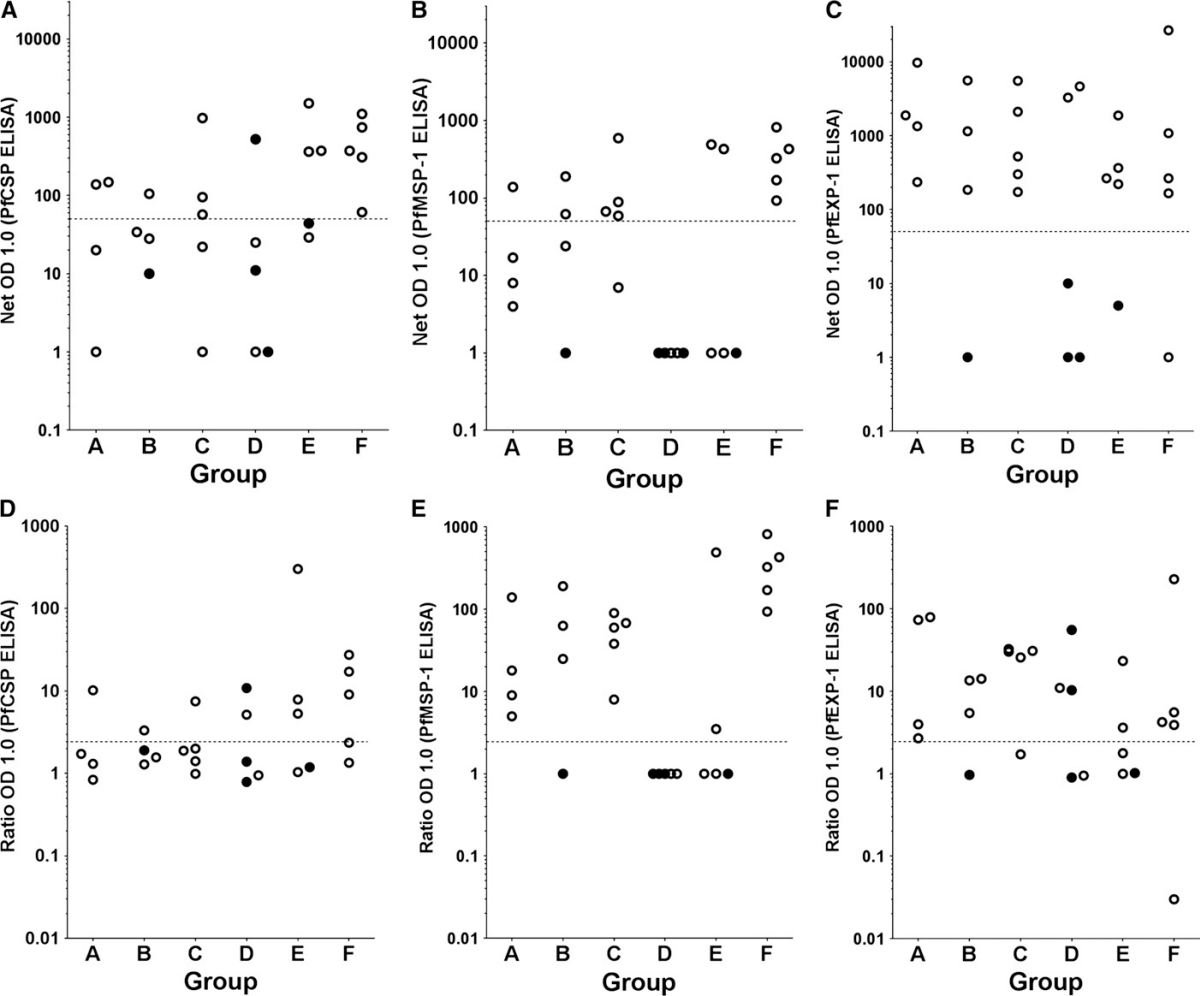
Depicted are antibodies expressed as net optical density (OD) (**A**–**C**) and ratio OD (**D**–**F**) to *Plasmodium falciparum* circumsporozoite protein (PfCSP), *P. falciparum* exported protein 1 (PfEXP1), and *P. falciparum* merozoite surface protein 1 (PfMSP1) by enzyme-linked immunosorbent assay (ELISA) in sera collected 28 days after administration of *P. falciparum* sporozoite (PfSPZ) Challenge and stratified by group (Group A [10,000 PfSPZ, 2 × 50 μL], B [10,000 PfSPZ, 8 × 50 μL], C [10,000 PfSPZ, 2 × 10 μL], D [10,000 PfSPZ, 8 × 10 μL], E [50,000 PfSPZ, 2 × 10 μL], and F [50,000 PfSPZ, 8 × 10 μL]). The dotted line represents the threshold end point titer ≥ 50, which is considered to be indicative of antibody production. The closed circles represent individuals who did not develop parasitemia. Note: Sera were missing from one aparasitemic volunteer in Group B.

Antibodies to PfCSP (expressed in sporozoite and early liver stages), PfEXP1 (expressed in early, mid, and late liver, and asexual erythrocytic stages), and PfMSP1 and PfEBA-175 (expressed in late liver and asexual erythrocytic stages) were assessed by ELISA in sera collected on the day of injection of PfSPZ Challenge and 28 days later. An individual was arbitrarily considered to have had development of antibodies to a specific antigen if the difference between the OD 1.0 (serum dilution at which the optical density was 1.0) on Day 28 and the OD 1.0 before injection of PfSPZ Challenge (net OD 1.0) was ≥ 50 and the ratio of OD 1.0 on Day 28 to the OD 1.0 before injection (OD 1.0 Ratio) was ≥ 2.5. The results are summarized in Supplemental Table 2, Table 5, and Figure 1A–F

. None of the volunteers developed antibodies to PfEBA-175, but antibodies were developed to PfCSP, PfEXP1, and PfMSP1. The greatest percentage of responders was to PfEXP1 (68%), as was the highest net OD 1.0 among responders (26,748). However, the highest OD 1.0 ratios were to PfMSP1. Only volunteers who developed *P. falciparum* parasitemia developed antibodies to PfMSP1 or PfEXP1, but one volunteer who did not develop parasitemia developed antibodies to PfCSP. There was no significant difference in antibodies to PfEXP1, or PfMSP1 in volunteers who received 1 × 10^4^ versus 5 × 10^4^ PfSPZ. However, there was a significantly higher net OD 1.0 against PfCSP in those who received 5 × 10^4^ PfSPZ (GM net OD 1.0 of 261 versus 24, *P* = 0.003, Mann–Whitney *U* test) and the difference in OD 1.0 ratios in the 5 × 10^4^ versus 10^4^ PfSPZ groups approached statistical significance (GM 6.8 versus 2.1, *P* = 0.07). The IFA results were consistent with the ELISA results (see Supplemental Table 1 and Supplemental Figure 1A and B). Antibody results did not correlate with the magnitude of qPCR.

### CQ and DCQ kinetic sub-study.

**Figure 2.**
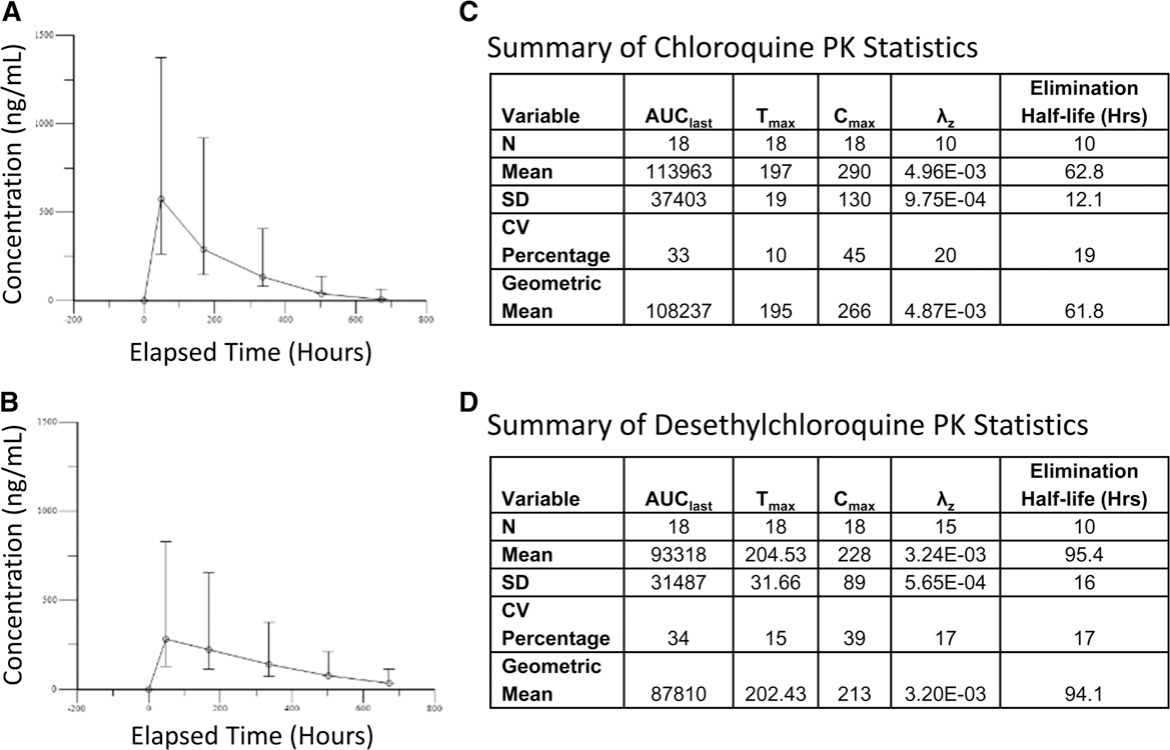
Depicted is the line plot of chloroquine (CQ) (**A**) and desethylchloroquine (DCQ) (**B**) concentration by nominal time after treatment dosing with CQ at hours 0, 6, 24, and 48. A summary of CQ (**C**) and DCQ (**D**) pharmacokinetics (PK) is presented with area under the concentration-time curve from time zero to final sample (AUC_max_), time of maximum concentration (T_max_), maximum concentration (C_max_), slope of the elimination phase (λ_z_), standard deviation (SD), and coefficient of variation (CV) depicted.

Blood and urine drug concentrations were collected from all 18 subjects at all six time points. The demographic details of the volunteers included age range from 21 to 44 years and weight range from 59 to 118 kg, with a median weight of 79 kg. Twelve (67%) of the 18 subjects were male and each group (A–F) had between two and four representative participants. CQ and DCQ concentrations were assessed from blood samples. The combined concentration of CQ and DCQ was assessed from the urine samples. The LLQs were 51 ng/mL for blood CQ, 43 ng/mL for blood DCQ, and 1.25 μg/mL for urine combined CQ and DCQ. All Day 0 concentrations were below the LLQ. Figure 2A and B

presents line plots for the CQ and DCQ blood concentration distributions by time. Concentrations were the highest on Day 2 for both CQ and DCQ. Concentrations tended to decrease slower for DCQ than CQ. Eighteen of 18 (100%) volunteers had CQ and DCQ levels above the LLQ at hour 336 (Day 14 post-malaria episode). Ten (56%) subjects had Day 21 post-malaria episode (nominal hour 504) and 2 (11%) subjects had Day 28 post-malaria episode (nominal hour 672) CQ concentrations above the LLQ. Sixteen (89%) participants had Day 21 and 11 (61%) participants had Day 28 DCQ concentrations that were above the LLQ. No subject had a Day 28 CQ or DCQ blood concentration above the LLQ after Day 21 concentrations below the LLQ. The relationship between CQ concentration and parasite clearance cannot be established from these data, because 16 (89%) of the 18 subjects cleared the parasite before the first post-baseline PK sample.

CQ PK summary statistics are presented in Figure 2C. The value of λ_z_ could not be estimated in eight subjects due to insufficient samples above the LLQ. All subjects had a time of maximum concentration (T_max_) associated with the Day 7 post-malaria episode (nominal 168 hours) sample; subject maximum concentrations (C_max_) at this time ranged from 144 to 628 ng/mL. The mean C_max_ was 290 ng/mL with a coefficient of variation (CV) of 45%. The mean elimination half-life was 62.8 hours (2.6 days) with a CV of 19%.

Subject-level DCQ PK parameters are listed in Figure 2D. The value of λ_z_ could not be estimated in three subjects due to insufficient samples above the LLQ or a low R2 value. Seventeen (94%) of the 18 subjects had a T_max_ associated with the 7-day sample; C_max_ concentrations ranged from 112 to 432 ng/mL. Mean C_max_ was 228 ng/mL with a CV of 39% and the mean elimination half-life was 95.4 hours (4 days) with a CV of 17%.

Combined CQ and DCQ urine concentrations reveal that 14 (78%) of the 18 participants had data above the LLQ at the final collection time. One additional participant had a sample below the LLQ on Day 21 but above the LLQ on Day 28. The maximum-recorded concentration was 262 μg/mL on Day 2. The maximum concentration recorded after the last dose of drug was 52.8 μg/mL on Day 7 (data not shown).

## Discussion

ID administration of infectious PfSPZ by needle and syringe was safe and conferred malaria to 100% of recipients in three of the six groups. The levels of reactogenicity and safety of the PfSPZ product administered by ID inoculation by needle and syringe were similar, if not less than when *P. falciparum* malaria is conferred by infected mosquito bite. Previous to this study, investigators demonstrated 83% infectivity of 2,500; 10,000; or 25,000 PfSPZ administered by ID inoculation in two aliquots of 50 μL.^5^,^18^ These results were validated in African volunteers.^6^ We sought to improve on the efficiency of PfSPZ infectivity by analyzing the effect of aliquot, volume, and PfSPZ quantity. Contemporaneously with the conduct of this study, IM^18^ and IV^19^ inoculations were shown to be highly successful.

To mimic mosquito via ID injection, we chose to weight our trial design to smaller injectate reasoning that the sporozoites might be trapped in larger injectate volumes, thereby minimizing capillary penetration. Of the six groups tested (Groups A–F), three demonstrated 100% infectivity in the transmission of *P. falciparum* malaria (Group A: 4/4 subjects (1 withdrawal), 1 × 10^4^ PfSPZ in 50 μL/2 doses; Group C: 5/5 subjects, 1 × 10^4^ PfSPZ in 10 μL/2 doses; and Group F: 5/5 subjects, 5 × 10^4^ PfSPZ in 10 μL/8 doses). We did not find a clear association with either aliquot volume or PfSPZ quantity, but this may be reflective of the limitations of the needle and syringe administration. The mosquito proboscis is able to probe in an extremely efficient manner, ensuring delivery of PfSPZ to capillaries and into the circulation.^20^ This might also explain why IV PfSPZ has been proven to be so successful.^19^ Whether microinjection devices could improve on the efficiency of PfSPZ administration remains to be tested. The mechanics of PfSPZ administration are critically important for PfSPZ Challenge, but also apply to administration of the promising PfSPZ vaccine, which is composed of identical PfSPZ that have been radiation attenuated.^15^,^21^

The efficiency of PfSPZ to enter circulation may also influence the kinetics of malaria infection. The overall prepatent period by blood smear in this study was a mean of 13.5 days (range 12–16) and the shortest prepatent period was 12.7 days. This was more than the 10.9 days we have seen in CHMI administered by the bite of mosquitoes,^10,11^ but consistent with what has been noted in prior PfSPZ Challenge by the ID and IM routes. qPCR successfully detected parasitemia with a mean of 10.7 days after CHMI (range 8.4–11.9). This was longer than the 6.5 days seen in the mosquito bite studies.^10,11^ The growth rate in the blood appeared similar to traditional CHMI. Thus, this prolonged prepatent period almost certainly reflected the numbers of PfSPZ that successfully traveled from the skin to the liver, invaded hepatocytes, and fully developed to blood stage.

On examination of the individual groups, Group F was deemed to have the most parameters aligned with the traditional CHMI administered by the bite of mosquito (prepatent period = 12.7 days [range 12–14 days]). The group sample sizes were limited to five individuals, for practical reasons of malaria management, and capped at a total study size of 30 volunteers. Although this was not powered to achieve statistical significance, CHMI is a potent technique with a discrete outcome measure of “infected” or “not infected.” Sample sizes of five or six individuals are commonly used as controls in CHMI in which 100% infectivity would be expected. Hence, departure from this expected outcome would be a reliable study outcome. The clinical signs and symptoms associated with mosquito-transmitted malaria were also most similar in the volunteers who received 5 × 10^4^ PfSPZ (Groups E and F). Group D: 2/5 subjects, 1 × 10^4^ PfSPZ in 10 μL/8 doses had the lowest prevalence of malaria transmission (prepatent period = 13.0 days). Thus, in regard to prepatent period, our results with 5 × 10^4^ PfSPZ were similar to those reported from the Netherlands with fewer PfSPZ, and were consistent with the fact that no dose response was seen in that study.

Signs and symptoms associated with administration of PfSPZ Challenge included mild and self-resolving local reactions. Symptoms associated with malaria included systemic abnormalities including severe fever, chills, and malaise, most predominant in volunteers receiving 5 × 10^4^ PfSPZ. These symptoms were expected and transient in nature, resolving on malaria therapy. In addition, transient laboratory abnormalities associated with clinical malaria were noted, characterized by rises in AST, ALT, bilirubin, and creatinine and transient declines in hemoglobin and white blood cells in some subjects. These laboratory abnormalities were within the expected range of findings associated with mild *P. falciparum* malaria and were evenly distributed across all groups. All abnormalities resolved without sequelae.

An isolated finding of a transient positive qPCR after the diagnostic study period is of questionable significance. First, qPCR was a research modality and not a widely accepted means of diagnosis at the time of this study. The presence of gametocytes might be expected after treatment at Day 28 post-CHMI (13 days following therapy) and could account for transient low-level qPCR positivity.^22^ Gametocytes being largely resistant to most anti-malarial treatment can persist within the circulation weeks after treatment.^23^ The qPCR detection self-resolved before administration of any anti-malarial product, and the malaria culture (the most sensitive detection method) remained negative at 4 weeks. Importantly, the subject remained asymptomatic and blood smear negative throughout follow-up evaluation.

The antibody findings are important in that they show the antibody profile 4 weeks after a single exposure to PfSPZ and ∼2 weeks after initiation of successful treatment of a single, low parasite density parasitemia. About 36% of subjects developed low levels of antibodies to the sporozoite and early liver stage antigen, PfCSP. Interestingly, the incidence rate of seroconversion and the magnitude of response were related to the dose. In those who received 1 × 10^4^ PfSPZ, 4/18 (22%) seroconverted and among those who received 5 × 10^4^ PfSPZ, 6/10 (60%) (*P* = 0.09, Fisher's exact test, 2 tailed). The magnitude of net OD 1.0 was ∼10 times higher in the 5 × 10^4^ PfSPZ groups (GM net OD 1.0 of 261 versus 24, *P* = 0.003, Mann–Whitney *U* test). The responses to PfEXP1 and PfMSP1 were greater than to PfCSP, and almost certainly reflect exposure to these antigens during the ∼8 days from initial release of parasites from the liver (∼Day 5.5) to initiation of treatment (Day 13.5), as the five individuals who did not develop parasitemia did not develop antibodies to these two antigens (Figure 1). The incidence rate of seroconversion to PfEXP1 (68%) was not significantly different from that to PfMSP1 (50%). However, the magnitude of net OD 1.0 was higher for PfEXP1 and the ratio of post- to pre-net OD 1.0 was higher for PfMSP1. It is unclear as to why antibodies did not develop to PfEBA-175. The IFA results were also consistent with ELISA results (see Supplemental Tables 1 and 2 and Supplemental Figure 1A and B).

CQ and DCQ levels (sera and urine) were calculated to determine effective dose levels and drug kinetics during the treatment phase of the study. Samples were collected at all six time points to ascertain a kinetic profile of drug detection after a single treatment regimen (1,500 mg CQ base/2,500 mg salt as malaria treatment over 48 hours). It appears, based on these data, that a single CQ dosing regimen results in detectable CQ and DCQ levels above the LLQ (51 and 43 ng/mL, respectively) out to Day 14 post-administration in all 18 volunteers tested; however, the assay was limited in quantification of levels below the LLQ, which was 10-fold greater than the minimal inhibitory concentration biologically required to inhibit parasite growth (5 ng/mL). These results may prove valuable in optimizing directly observed therapy in the PfSPZ-CVac model of injecting non-irradiated, fully infective PfSPZ serially under cover of anti-malarial prophylaxis as a means of achieving full protection against malaria.

In conclusion, ID administration of aseptic, purified, cryopreserved, infectious PfSPZ (PfSPZ Challenge) by needle and syringe was safe and transmitted malaria to 100% of human subjects in three of the six groups. About 100% infection with the shortest prepatent period was achieved in the group that received the highest dose (5 × 10^4^ PfSPZ) in the most inoculations (*N* = 8), suggesting that a further reduction in the prepatent period should come from increasing the numbers of PfSPZ and inoculations. Administering so many different inoculations by needle and syringe may prove cumbersome, so microneedle array injection is being considered. However, the report that IV or direct venous inoculation of far fewer PfSPZ achieves 100% infection with a prepatent period of 11.3 days^19^ establishes a target level of infection with a target number of PfSPZ for those working to achieve similar results by ID injection, a result that may prove difficult to achieve.

## Supplementary Material

Supplemental Datas.

## Figures and Tables

**Table 1:** Demographics and randomization of study participants

PfSPZ Challenge group	Dose of PfSPZ	Aliquot volume (μL)	PfSPZ/μL/injection	Number of injections	Number of Subjects	Age and range	Gender (no. of female)
A: Medium dose, medium aliquot volume*	10,000	50	100	2	5	35.6 (30–43)	3/5
B: Medium dose, medium aliquot volume	10,000	50	25	8	5	38.2 (28–44)	0/5
C: Medium dose, low aliquot volume	10,000	10	500	2	5	36.6 (21–44)	0/5
D: Medium dose, low aliquot volume	10,000	10	125	8	5	31.6 (22–44)	1/5
E: High dose, low aliquot volume	50,000	10	2,500	2	5	29.2 (24–33)	2/5
F: High dose, low aliquot volume	50,000	10	625	8	5	27.8 (24–32)	5/5

PfSPZ = *Plasmodium falciparum* sporozoite; RUNMC = Radboud University Nijmegen Medical Center.

* Group A represents a bridging or reference arm to a previous study performed at RUNMC.^5^

**Table 2:** Maximum intensity of solicited symptoms and signs during post-CHMI after receiving *Plasmodium falciparum* sporozoites by ID injection or by traditional mosquito (first 48 hours of local reactogenicity discounted due to expected reactions associated with mosquito bites)

All groups combined		Post-ID CHMI (*N* = 30)				Post-traditional CHMI (days 2–7) (*N* = 25)			
	Reactogenicity	None *n* (%)	Mild *n* (%)	Moderate *n* (%)	Severe *n* (%)	None *n* (%)	Mild *n* (%)	Moderate *n* (%)	Severe *n* (%)
Systemic	Oral temperature	30 (100.0)	0	0	0	22 (88.0)	3 (12.0)	0	0
(Day 0–7)	Malaise	19 (63.3)	9 (30.0)	2 (6.7)	0	18 (72.0)	5 (20.0)	2 (8.0)	0
	Nausea	27 (90.0)	2 (6.7)	1 (3.3)	0	23 (92.0)	2 (8.0)	0	0
	Myalgia	21 (70.0)	4 (13.3)	5 (16.7)	0	25 (100.0)	0	0	0
	Headache	22 (73.3)	4 (13.3)	4 (13.3)	0	22 (88.0)	2 (8.0)	1 (4.0)	0
	Chills	27 (90.0)	3 (10.0)	0	0	25 (100.0)	0	0	0
	Vomiting	29 (96.7)	1 (3.3)	0	0	25 (100.0)	0	0	0
	Any systemic	17 (56.7)	7 (23.3)	6 (20.0)	0	6 (20.7)	9 (31.0)	11 (37.9)	3 (10.3)
Local	Pain	24 (80.0)	6 (20.0)	0	0	23 (92.0)	1 (4.0)	0 (0)	0 (0)
(Day 2–14)	Tenderness	26 (86.7)	3 (10.0)	1 (3.3)	0	N/A	N/A	N/A	N/A
	Erythema	28 (93.3)	1 (3.3)	1 (3.3)	0	13 (52.0)	10 (40.0)	2 (8.0)	1 (4.0)
	Induration	30 (100.0)	0	0	0	17 (68.0)	7 (28.0)	0 (0)	1 (4.0)
	Any local	22 (73.3)	6 (20.0)	2 (6.7)	0	12 (48.0)	10 (40.0)	2 (8.0)	1 (4.0)
Any	Any symptoms	14 (46.7)	8 (26.7)	8 (26.7)	0	4 (16.0)	15 (60.0)	3 (12.0)	2 (8.0)

CHMI = controlled human malaria infection; ID = intradermal.

**Table 3:** Maximum intensity of solicited symptoms and signs associated during inpatient days 8–18 (during which time malaria is expected) and stratified by volunteers who received ID challenge, those who received the high dose PfSPZ exposure of 50,000 and historical results from mosquito-transmitted (i.e., traditional) CHMI

Inpatient stay (days 8–18)	ID CHMI all groups (A–F) (*N* = 29)				ID CHMI with 5 × 10^4^ PfSPZ (E, F) (*N* = 10)				Traditional challenge—mosquito (*N* = 25)				*P* value ID vs. traditional*
Reactogenicity	None *n* (%)	Mild *n* (%)	Moderate *n* (%)	Severe *n* (%)	None *n* (% of total)	Mild *n* (% of total)	Moderate *n* (% of total)	Severe *n* (% of total)	None *n* (%)	Mild *n* (%)	Moderate *n* (%)	Severe *n* (%)	
Oral temperature	15 (51.7)	3 (10.3)	9 (31.0)	2 (6.9)	2 (13.3)	0	6 (66.7)	2 (100.0)	6 (24.0)	3 (12.0)	5 (20.0)	11 (44.0)	**0.037**
Malaise	11 (37.9)	9 (31.0)	7 (24.1)	2 (6.9)	0	5 (56.0)	4 (57.1)	1 (50.0)	6 (24.0)	10 (40.0)	9 (36.0)	0	0.28
Nausea	17 (58.6)	11 (37.9)	1 (3.4)	0	3 (17.6)	6 (54.5)	1 (100.0)	0	13 (52.0)	11 (44.0)	1 (4.0)	0	0.63
Myalgia	15 (51.7)	7 (24.1)	7 (24.1)	0	1 (0.07)	4 (57.1)	5 (71.4)	0	7 (28.0)	11 (44.0)	7 (28.0)	0	0.13
Headache	12 (41.4)	12 (41.4)	4 (13.8)	1 (3.4)	2 (16.6)	3 (25.0)	0	0	3 (12.0)	15 (60.0)	7 (28.0)	0	**0.017**
Chills	18 (62.1)	4 (13.8)	6 (20.7)	1 (3.4)	4 (22.2)	1 (25.0)	4 (66.7)	1 (100.0)	5 (20.0)	11 (44.0)	9 (36.0)	0	**0.002**
Vomiting	25 (86.2)	4 (13.8)	0	0	7 (28.0)	3 (75.0)	0	0	21 (84.0)	4 (16.0)	0	0	ns
Abdominal pain	19 (65.5)	9 (31.0)	1 (3.4)	0	7 (36.8)	2 (22.2)	1 (100.0)	0	24 (96.0)	0	1 (4.0)	0	ns
Diarrhea	25 (86.2)	3 (10.3)	1 (3.4)	0	7 (28.0)	2 (66.7)	1 (100.0)	0	23 (92.0)	2 (8.0)	0	0	ns
Dizziness	24 (82.8)	4 (13.8)	1 (3.4)	0	8 (33.3)	1 (25.0)	1 (100.0)	0	13 (52.0)	9 (36.0)	3 (12.0)	0	ns
Arthralgia	20 (69.0)	4 (13.8)	5 (17.2)	0	5 (25.0)	2 (50.0)	3 (60.0)	0	16 (64.0)	4 (16.0)	5 (20.0)	0	ns
Chest pain	26 (89.7)	3 (10.3)	0	0	9 (34.6)	1 (33.3)	0	0	22 (88.0)	3 (12.0)	0	0	ns
Shortness of breath	25 (86.2)	4 (13.8)	0	0	8 (32.0)	2 (50.0)	0	0	22 (88.0)	3 (12.0)	0	0	ns
Change in exercise Tolerance	26 (89.7)	1 (3.4)	2 (6.9)	0	5 (19.2)	0	0	0	20 (80.0)	4 (16.0)	1 (4.0)	0	ns
Palpitations	27 (93.1)	2 (6.9)	0	0	5 (18.5)	0	0	0	ND	ND	ND	ND	ND
Any systemic	6 (20.7)	9 (31.0)	11 (37.9)	3 (10.3)	0	2 (22.2)	6 (54.5)	2 (66.7)	0	5 (20.0)	9 (36.0)	11 (44.0)	**0.025**

CHMI = controlled human malaria infection; ID = intradermal; PfSPZ = *Plasmodium falciparum* sporozoite. Bold values represent those calculations that reached statistical significance.

* *P* value determined by Mantel–Haenszel Chi^2^ analysis (no symptoms vs. symptoms) or Fishers exact (2 tailed) as appropriate between ID CHMI (all groups) and traditional CHMI.

**Table 4:** Results and characteristics of malaria stratified by group

Study group	Patent volunteers (*n*)	Prepatent period (days) (range)*	Smear parasite density (μL)*	Time (hours) to first PCR (days)*	First PCR positive (para/μL)*	PCR (para/μL) at patency*
A: 1 × 10^4^ PfSPZ 2 × 50 μL	4/4†	14.5 (13–16)	14.7 (4–99)	286.6 (11.9)	0.156	18.3
B: 1 × 10^4^ PfSPZ 8 × 50 μL	3/5	14.0 (14)	46.3 (25–78)	258.6 (10.8)	0.549	64.4
C: 1 × 10^4^ PfSPZ 2 × 10 μL	5/5	13.5 (13–14)	8.1 (2–134)	259.6 (10.8)	0.210	17.8
D: 1 × 10^4^ PfSPZ 8 × 10 μL	2/5	13.0 (13)	10.5 (10–11)	201 (8.4)	0.050	17.2
E: 5 × 10^4^ PfSPZ 2 × 10 μL	4/5	13.2 (12–15)	10.2 (5–15)	239.7 (10.0)	0.161	24.8
F: 5 × 10^4^ PfSPZ 8 × 10 μL	5/5	12.7 (12–14)	21.4 (8–46)	247.6 (10.3)	0.154	25.6
Combined groups	23/29	13.5 (12–16)	15.0 (2–134)	256.8 (10.7)	0.161 (0.015–6.16)	24.5 (6.8–105)

CHMI = controlled human malaria infection; PCR = polymerase chain reaction; PfSPZ *= Plasmodium falciparum* sporozoite.

* Blood smear, time, and PCR quantity expressed as geometric mean PCR parasite density. Prepatent period is expressed as mean.

† Denominator reflects withdrawal of volunteer after CHMI (data from two volunteers removed due to missing interval sample).

**Table 5:** Antibodies to PfCSP, PfEXP1, and PfMSP1 by ELISA in sera collected 28 days after administration of PfSPZ Challenge as compared with in preinjection sera

Group	PfCSP					PfEXP1					PfMSP1				
	Net OD 1.0 ≥ 50 (±total)	Mean net OD 1.0 of positives	OD 1.0 ratio ≥ 2.5 (±total)	Mean OD 1.0 ratio of positives	Overall positives (±total)*	Net OD 1.0 ≥ 50 (±total)	Mean net OD 1.0 of positives	OD 1.0 ratio ≥ 2.5 (±total)	Mean OD 1.0 ratio of positives	Overall positives (±total)*	Net OD 1.0 ≥ 50 (±total)	Mean net OD 1.0 of positives	OD 1.0 ratio ≥ 2.5 (±total)	Mean OD 1.0 ratio of positives	Overall positives (±total)*
A	2/4	143	1/4	10.2	1/4	4/4	3,302	4/4	40.0	4/4	1/4	139	4/4	43.0	1/4
B	1/4	105	1/4	3.3	1/4	3/4	2,306	3/4	11.1	3/4	2/4	126	3/4	93.0	2/4
C	3/5	376	1/5	7.5	1/5	5/5	1,728	4/5	29.9	4/5	4/5	202	5/5	52.8	4/5
D	1/5	523	2/5	8.0	1/5	2/5	3,964	3/5	27.7	2/5	0/5	1	0/5	-	0/5
E	3/5	746	3/5	105.0	3/5	4/5	683	2/5	13.5	2/5	2/5	460	2/5	247.8	2/5
F	5/5	517	3/5	17.9	3/5	4/5	7,065	4/5	60.8	4/5	5/5	367	5/5	368.4	5/5
					10/28					19/28					14/28

PfCSP = *Plasmodium falciparum* circumsporozoite protein; PfEXP1 = *P. falciparum* exported protein 1; PfMSP1 = *P. falciparum* merozoite surface protein 1; PfSPZ *= P. falciparum* sporozoite; ELISA; enzyme-linked immunosorbent assay; OD = optical density.

* An individual was considered to have developed antibodies if the net OD 1.0 was ≥ 50 and the OD 1.0 ratio was ≥ 2.5 (see Methods for definitions).
