# Supplementary material for: Optimizing Intradermal Administration of Cryopreserved Plasmodium falciparum Sporozoites in Controlled Human Malaria Infection
Source: Am J Trop Med Hyg. 2015 Dec 9;93(6):1274–84. doi: 10.4269/ajtmh.15-0341 (PMC4674246; doi:10.4269/ajtmh.15-0341)
Supplement: Supplementary file 1 [file SD6.pdf]

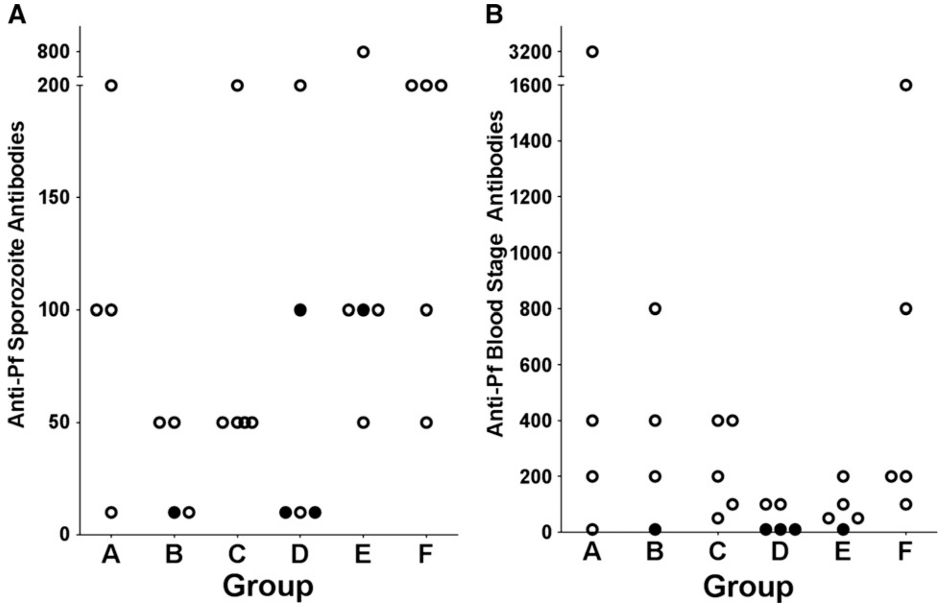

Supplemental Figure 1. End point titer antibodies to (**A**) *Plasmodium falciparum* sporozoite (PfSPZ) and (**B**) *P. falciparum* asexual erythrocytic stage parasites were assessed in sera taken 28 days after injection of PfSPZ Challenge and stratified by group (Groups A–D [10,000 PfSPZ], E and F [50,000 PfSPZ]). The last serum dilution at which the immunofluorescence assay (IFA) was positive (end point titer) was determined. An individual was considered to have developed antibodies to PfSPZ or blood stage parasites if the end point titer was ≥ 50; all results < 50 were arbitrarily graphed as 10. The results for the individuals who developed parasitemia are shown as open circles (N = 23), and those who did not develop parasitemia (N = 5) are shown as closed circles. Note: y axis scale varies for antibody.

Supplemental Table 1

End point titer antibodies to PfSPZ and Pf asexual erythrocytic stage parasites by IFA for each volunteer from sera collected 28 days after administration of PfSPZ Challenge, showing the last serum dilution at which the IFA was positive. Preinjection sera for all volunteers were negative

	Anti-PfSPZ		Anti-Pf asexual erythrocytic stage parasites	
Group	End point titer ≥ 50 (±total)	Geometric mean of positives*	End point titer ≥ 50 (±total)	Geometric mean of positives*
A	3/4	126	3/4	635
B	2/4	50	3/4	400
C	5/5	66	5/5	174
D	2/5	141	2/5	100
E	5/5	132	4/5	84
F	5/5	132	5/5	348
	**22/28**		**22/28**	

IFA = immunofluorescence assay; PfSPZ = *Plasmodium falciparum* sporozoite; Pf = *P. falciparum.*
*An individual was considered positive if the end point titer was ≥ 50 (see Methods for definitions).

Supplemental Table 2

Antibody titers to PfSPZ as measured by ELISA and Pf asexual erythrocytic stage parasites by IFA for each volunteer from sera collected preinection (Visit 1) and 28 days (Visit 20) after administration of PfSPZ Challenge

			Antibody titers measured by ELISA				Antibody titers measured by IFA	
Subject ID	Group	Visit no.	PfCSP	PfEBA-175	PfMSP-1	PfEXP-1	Sporozoite IFA	Blood stage IFA
M11UMD002	C	1	64	1	1	72	< 50	< 50
M11UMD002		20	121	1	90	2,183	50	400
M11UMD006	A	1	15	2	1	24	< 50	< 50
M11UMD006		20	153	1	140	1,906	200	400
M11UMD007	A	1	203	1	1	451	< 50	< 50
M11UMD007		20	351	1	18	1,796	100	200
M11UMD008	D	1	758	1	1	351	< 50	< 50
M11UMD008		20	720	1	1	3,630	< 50	100
M11UMD012	C	1	150	1	16	175	< 50	< 50
M11UMD012		20	1,125	1	609	5,709	200	400
M11UMD014	B	1	11	6	1	29	< 50	< 50
M11UMD014		20	21	20	1	28	< 50	< 50
M11UMD015	E	1	5	1	1	84	< 50	< 50
M11UMD015		20	1,509	1	492	1,964	800	200
M11UMD017	C	1	95	1	1	10	< 50	< 50
M11UMD017		20	190	1	68	310	50	50
M11UMD018	E	1	235	1	1	252	< 50	< 50
M11UMD018		20	279	1	1	257	100	< 50
M11UMD032	C	1	54	1	1	21	< 50	< 50
M11UMD032		20	76	1	8	542	50	100
M11UMD035	F	1	45	1	1	117	< 50	< 50
M11UMD035		20	106	1	820	26,865	200	800
M11UMD036	B	1	117	1	1	258	< 50	< 50
M11UMD036		20	151	1	25	1,407	50	800
M11UMD039	D	1	6	1	1	85	< 50	< 50
M11UMD039		20	31	5	1	4,733	200	100
M11UMD040	B	1	45	1	1	444	< 50	< 50
M11UMD040		20	150	1	191	6,028	50	400
M11UMD046	D	1	33	4	1	31	< 50	< 50
M11UMD046		20	26	5	1	28	< 50	< 50
M11UMD047	E	1	786	7	1	470	< 50	< 50
M11UMD047		20	815	5	1	836	50	50
M11UMD054	D	1	28	1	1	1	< 50	< 50
M11UMD054		20	39	1	1	11	< 50	< 50
M11UMD057	B	1	49	8	1	14	< 50	< 50
M11UMD057		20	77	1	63	199	< 50	200
M11UMD060	F	1	883	4	1	333	< 50	< 50
M11UMD060		20	1,191	1	327	1,413	100	200
M11UMD063	C	1	101	1	1	241	< 50	< 50
M11UMD063		20	100	1	60	414	50	200
M11UMD064	E	1	84	1	1	84	< 50	< 50
M11UMD064		20	447	1	1	305	100	50
M11UMD068	F	1	46	1	1	58	< 50	< 50
M11UMD068		20	418	3	430	323	50	100
M11UMD071	F	1	28	1	1	33	< 50	< 50
M11UMD071		20	770	1	94	1	200	1,600
M11UMD072	E	1	54	14	12	1	< 50	< 50
M11UMD072		20	426	10	42	1	100	100
M11UMD073	D	1	53	1	1	37	< 50	< 50
M11UMD073		20	576	1	1	35	100	< 50
M11UMD077	F	1	68	1	1	57	< 50	< 50
M11UMD077		20	1,169	10	171	223	200	200
M11UMD079	A	1	64	1	1	138	< 50	< 50
M11UMD079		20	84	1	5	373	< 50	< 50
M11UMD088	A	1	125	1	1	134	< 50	< 50
M11UMD088		20	105	1	9	9,879	100	3,200

ELISA = enzyme-linked immunosorbent assay; IFA = immunofluorescence assay; PfCSP = *Plasmodium falciparum* circumsporozoite protein; PfEBA-175 = *P. falciparum* erythrocyte-binding antigen-175; PfEXP1 = *P. falciparum* exported protein 1; PfMSP1 = *P. falciparum* merozoite surface protein 1; PfSPZ = *P. falciparum* sporozoite.

Sera from M11UMD016 (Group B) is missing from analysis. The Group designation refers to dose, number of injections, and volume of injection: Group A (10,000 PfSPZ, 2 × 50 μL), B (10,000 PfSPZ, 8 × 50 μL), C (10,000 PfSPZ, 2 × 10 μL), D (10,000 PfSPZ, 8 × 10 μL), E (50,000 PfSPZ, 2 × 10 μL), and F (50,000 PfSPZ, 8 × 10 μL).
